# Establishment of a Large‐Scale PDX Library of Head and Neck Cancers for Functional Precision Oncology

**DOI:** 10.1002/cam4.71521

**Published:** 2026-02-18

**Authors:** Hisano Yanagi, Tomoki Kuki, Tetsuya Takimoto, Miki Takabayashi, Seiji Yamada, Chikako Yagi, Yoshitake Kiryu, Maki Kurimoto, Miho Ishikawa, Arisa Yoshida, Yasuyoshi Mizutani, Atsushi Enomoto, Motoshi Suzuki, Kenji Kawada, Hisayuki Kato, Ichiro Tateya, Hideyuki Saya, Takashi Watanabe

**Affiliations:** ^1^ Department of Medical Oncology Fujita Health University School of Medicine Toyoake Japan; ^2^ Oncology Innovation Center Fujita Health University Toyoake Japan; ^3^ Department of Otolaryngology‐Head and Neck Surgery Fujita Health University School of Medicine Toyoake Japan; ^4^ Department of Pathology Nagoya University Graduate School of Medicine Nagoya Japan; ^5^ Department of Molecular Oncology Fujita Health University School of Medicine Toyoake Japan

**Keywords:** chemotherapy sensitivity, functional precision oncology, head and neck cancer, patient‐derived xenograft (PDX) library, preclinical mouse model

## Abstract

**Background:**

Precision oncology leverages the molecular and genetic characteristics of tumors to enable accurate diagnosis and effective treatment selection. However, recent clinical trials have highlighted the limitations of current approaches and underscored the need to integrate static molecular profiling with functional analyses using patient‐derived xenograft (PDX) models—particularly for cancers such as head and neck cancer (HNC), where driver mutations are rare and prognosis remains poor.

**Methods:**

Here, we aimed to establish a large‐scale PDX library for HNC, termed the Fujita Xenograft Library (FXeL), annotated with detailed clinical information. Since 2022, tumor specimens from over 100 surgical cases at Fujita Health University Hospital have been transplanted into immunodeficient mice, resulting in the successful establishment of 62 PDX models.

**Results:**

Advanced clinical stage was significantly associated with successful engraftment, and serial passaging led to progressively accelerated tumor growth. Comparative analyses of genomic profiles between patient tumors and PDXs demonstrated that major cancer‐related mutations were largely preserved in PDXs, while clonal selection and evolution occurred during engraftment. Histopathological features, including keratinization and nuclear atypia, were retained, whereas stromal components such as cancer‐associated fibroblasts exhibited compositional shifts. Furthermore, drug sensitivity assays revealed that PDX responses to cisplatin (CDDP) closely mirrored the clinical outcomes of the corresponding patients.

**Conclusions:**

The FXeL represents a robust and scalable platform for investigating HNC biology and therapeutic response. Despite limitations such as stromal remodeling and the absence of an immune microenvironment, these models provide valuable translational insights and support the advancement of functional precision oncology.

## Graphical abstract

FXeL (Fujita Xenograft Library), a large‐scale PDX library of head and neck cancers for functional precision oncology, we have established over 60 cases from 100 tumor specimens including rare cancers.

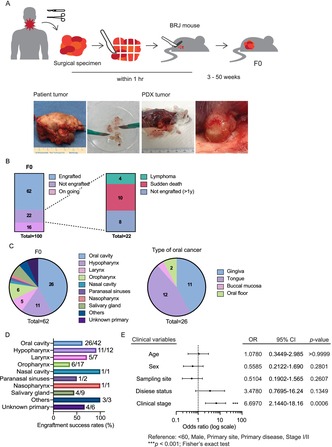

**Abbreviations**
APC: adenomatous polyposis coli
BRJ mice: Balb/c‐Rag2/Jak3 double‐deficient mice
CAF: cancer‐associated fibroblast
CDDP: *cis‐*diamminedichloroplatinum(II) (cisplatin)
FPO: functional precision oncology
FXeL: Fujita Xenograft Library
HE: hematoxylin and eosin
HNC: head and neck cancer
HPV: human papillomavirus
ISH: in situ hybridization
PDX: patient‐derived xenograft
SCC: squamous cell carcinoma
SNP: single nucleotide polymorphism
TME: tumor microenvironment
VAF: variant allele frequency

## 1 Introduction

Precision oncology, also referred to as personalized or tailored medicine, leverages the molecular and genetic characteristics of patient tumors to accurately diagnose cancer types and select effective treatment strategies [1]. Genomic analyses across various cancers have identified frequent genomic driver alterations, and testing for these alterations in individual tumors enables more effective, patient‐specific therapies [2]. Despite the promise of precision oncology, recent clinical trials—including the landmark National Cancer Institute's Molecular Analysis for Therapy Choice (NCI‐MATCH) trial—have underscored its inherent limitations. Notably, only a small fraction of patients (approximately 10%) derive clinical benefit from genomics‐guided therapies, and even among individuals with identical genetic alterations, therapeutic responses can vary markedly [3, 4]. Moreover, in certain cancers such as head and neck cancer (HNC) [5, 6] and triple‐negative breast cancer [7], mutations in tumor suppressor genes (e.g., TP53, CDKN2A) are frequently observed, whereas actionable driver alterations are seldom identified. These findings underscore the urgent need for integrative strategies that go beyond static genomic profiling.

Functional precision oncology (FPO) is an emerging approach that integrates descriptive genomic analyses with functional testing [4, 8]. Incorporating functional analyses provides a more comprehensive understanding of tumor biology and reveals actionable vulnerabilities in cancer cells [9]. Clinical specimens obtained from patients can be used for such assays using ‘live patient tumors’ [10, 11].

Patient‐derived xenograft (PDX) models are established by implanting tumor tissues from patients into immunodeficient hosts, and are known to preserve key genetic and histopathological features of the original tumors [12, 13, 14, 15]. Once established, PDXs can be maintained and serially passaged over time, offering reproducible and reliable models. Preclinical studies using PDXs have informed effective therapies by examining whether tumors harboring specific oncogenic mutations respond to targeted agents [13, 16]. Given the increasing need to connect genomic alterations with functional drug responses, PDX models have become indispensable platforms for FPO [4, 8, 9].

Previous studies using HNC PDX models have provided important insights into tumor biology and therapeutic response [17, 18, 19, 20]. Collectively, these reports have demonstrated associations between successful engraftment and aggressive clinical behavior, identified transcriptomic signatures predictive of patient outcomes, and revealed molecular determinants of drug sensitivity, such as responsiveness to cetuximab. Nevertheless, most of these investigations analyzed patient tumors or selected PDXs separately, rather than in matched patient–PDX pairs, and rarely incorporated in vivo therapeutic testing. Furthermore, few studies have integrated clinical information, genomic profiling, and pharmacological evaluation within a unified analytical framework.

To address these gaps, we established a large‐scale, clinically annotated PDX library for HNC—termed the Fujita Xenograft Library (FXeL)—using surgically resected tumor specimens from patients treated at Fujita Health University Hospital. FXeL uniquely integrates patient clinical data, pathological assessment, genomic profiling, and in vivo drug responses across matched patient–PDX pairs, providing a practical platform for FPO.

## 2 Materials and Methods

### 2.1 Patient Selection and Clinical Samples

Tumor specimens were obtained from patients diagnosed with HNC who underwent surgical treatment at Fujita Health University Hospital since June 2022. Surplus surgical tissues were used for this study. All participants were fully informed about the study and provided written informed consent prior to inclusion. The study protocol was approved by the Ethics Committee of Fujita Health University (HM24‐224). All animal experiments were conducted in accordance with the institutional guidelines of the Advanced Medical Research Center for Animal Models of Human Diseases at Fujita Health University (APU22132‐MD7).

### 2.2 Clinical Information

Clinical information, including age, sex, smoking history, and relevant medical history, was collected whenever available. Tumor stage was classified according to the Union for International Cancer Control (UICC) TNM classification. The summary of patient clinical characteristics is presented in Tables 1 and 2.

**TABLE 1:** Basic clinical information of patients (*n* = 100).

		range		median
Age		19–90		69.5
		**number**		**percentage**
Sex	Male		71	71%
	Female		29	29%
Recurrence	Primary		78	78%
	Recurrent		22	22%
Stage	I		17	17%
	II		16	16%
	III		19	19%
	IVA		33	33%
	IVB		8	8%
	IVC		1	1%
cancer of unknown			6	6%
TNM	*T*	T0	6	6%
		T1	14	14%
		T2	27	27%
		T3	24	24%
		T4	29	29%
	*N*	N0	43	43%
		N1	27	27%
		N2	22	22%
		N3	8	8%
	*M*	M0	98	98%
		M1	2	2%

*Note:* Of note, the TNM classification designated the cancer of unknown primary as T0.

**TABLE 2:** Location of patients' tumors (*n* = 100).

	number	percentage
Oral cavity	42	42%
Hypopharynx	12	12%
Larynx	7	7%
Oropharynx	17	17%
Nasal cavity	1	1%
Maxillary sinus	2	2%
Nasopharynx	1	1%
Salivary gland	9	9%
Others	3	3%
Unknown primary	6	6%

### 2.3 Generation of PDX and Passage

The surgeon excised surplus tissue (10–100 mm^3^) suspected to be cancerous from the surgical specimen and transferred it into D‐MEM/Ham's F‐12 medium (FUJIFILM Wako Pure Chemical Corporation) containing 100 μg/mL Primocin (Invivogen) and 5 μg/mL Cancidas (MSD). The tissue was kept on ice during transport to the animal facility. The tumor tissue was then transferred to a Petri dish in a biosafety cabinet and cut into approximately 2–30 mm^3^ pieces using a scalpel. The dissected tumor pieces were submerged in Matrigel (Corning) and subcutaneously transplanted into both flanks of Balb/c‐Rag2/Jak3 double‐deficient (BRJ) mice [21]. The BRJ mice were provided by Dr. Seiji Okada (Kumamoto University). The transplantation was typically completed within 1 h after tumor excision. The remaining tumor tissues were stored as described below. The mice were housed aseptically under a 12‐h light/12‐h dark cycle with free access to sterile food and water. Mice were anesthetized with a triple‐anesthetic mixture (medetomidine hydrochloride, Zenoaq; midazolam, Maruishi Pharmaceutical Co.; butorphanol tartrate, Meiji Seika Pharma) or isoflurane (Viatris) during invasive procedures.

Mice were monitored weekly for tumor growth. Mice engrafted with patient‐derived tumors were designated as F0, and tumors were serially passaged to subsequent generations (F1, F2, F3, etc.). Tumor passage was performed when the tumor volume reached 400–2000 mm^3^ or when a humane endpoint was met. Tumor volume was calculated using the formula: tumor volume (mm^3^) = (tumor length) (mm) × [tumor width (mm)]^2^/2, based on digital caliper measurements. Tumors were harvested from mice euthanized by cervical dislocation. Each tumor was cut into 2–30 mm^3^ fragments; a portion was immediately transplanted into new mice for the next passage, while the remainder was used for histopathological analysis or cryopreservation. Tumor tissues derived from patients or PDXs were stored in Cell Banker 1 solution (Zenogen Pharma) at −80°C overnight and then transferred to −150°C for long‐term storage.

### 2.4 Pathological Analysis

All pathological specimens were subjected to hematoxylin–eosin (HE) staining. The patient tumors were fixed in 10% neutral‐buffered formalin. PDX tumors were carefully dissected to remove surrounding non‐tumor tissue as much as possible and were fixed in 10% neutral‐buffered formalin at room temperature for 48–72 h. Formalin‐fixed, paraffin‐embedded (FFPE) blocks were prepared using an automated tissue processor. FFPE sections (3 μm thick) were cut and stained with HE using standard procedures. Lymphoma outgrowth in PDX mice was identified based on histopathological examination of HE‐stained sections.

### 2.5 In Situ Hybridization (ISH) Assay

ISH was performed as described previously [22]. Tissue sections were processed for RNA in situ detection using RNAscope 2.5 HD Reagent Kit–Brown (Advanced Cell Diagnostics) with the HybEZ II Hybridization System (Advanced Cell Diagnostics) according to the manufacturer's instructions. Human and mouse probes were hybridized to patient and PDX tumor sections, respectively. The probes used in this study were as follows: RNAscope probe–Hs‐ISLR, RNAscope probe–Mm‐Islr, RNAscope probe–Hs‐ACTA2‐O1, and RNAscope probe–Mm‐Acta2.

### 2.6 Targeted Exome Sequencing

Genomic profiling was performed in‐house using the *PleSSision‐Rapid‐Neo* platform as previously described [23]. Briefly, the tumor‐rich areas were identified and marked by pathologists on HE‐stained slides, and genomic DNA was extracted using the Maxwell RSC FFPE Plus DNA Kit (Promega). DNA quality and quantity were assessed prior to library preparation with the SureSelectXT Low Input Target Enrichment System with Pre‐Capture Pooling (Agilent Technologies). Target regions covering all 145 cancer‐related genes were captured using oligonucleotide probes and sequenced on the NextSeq 2000 instrument (Illumina). The mean and median on‐target sequencing depths across all samples were 500× and 441×, respectively.

### 2.7 Sequencing Read and Mutation Analysis

Sequencing data were processed using GenomeJack (Mitsubishi Electric Software, Tokyo, Japan), a commercially available bioinformatics pipeline [24, 25]. Briefly, sequencing reads were aligned to the human reference genome (UCSC hg19) using the Burrows–Wheeler Aligner (BWA) and realigned with ABRA to improve indel detection. For samples derived from PDXs, reads originating from the mouse genome were removed using XenofilteR [26] prior to variant calling to prevent host contamination.

Single‐nucleotide variants (SNVs) were identified using SAMtools, and sequence reads with fewer than 10 supporting counts or with conflicting signals between multiple amplicons were excluded. In addition, SNVs with a variant allele frequency (VAF) below 10% were disregarded as potential sequencing errors. Insertions and deletions (indels) were detected using a modified version of VarScan, with a significance cutoff of *p* < 0.05.

Putative germline single‐nucleotide polymorphisms (SNPs) were filtered using population databases, including dbSNP (https://www.ncbi.nlm.nih.gov/snp/), the Human Genetic Variation Database (HGVD) (http://www.hgvd.genome.med.kyoto‐u.ac.jp/index.html), and the ToMMo 2KJPN Database (https://ijgvd.megabank.tohoku.ac.jp; Tohoku Medical Megabank Organization, Sendai, Japan).

Mutation annotation was performed using multiple databases, including COSMIC (https://cancer.sanger.ac.uk/cosmic), ClinVar (https://www.ncbi.nlm.nih.gov/clinvar), CIViC (https://civicdb.org/home), SnpEff (https://pcingola.github.io/SnpEff), and Clinical Knowledgebase (CKB: https://ckb.jax.org). Copy‐number alterations (CNAs) were calculated as previously described [24].

### 2.8 Image Acquisition and Analysis

Brightfield microscopy images were acquired using a BX53 microscope (Olympus) equipped with 4×, 10×, 20×, or 40× objective lenses, a DP73 digital camera (Olympus), and CellSens software version 1.13 (Olympus). Image processing was performed using ImageJ/Fiji (version 2.9.0/1.54d).

### 2.9 Drug Sensitivity Analysis

For in vivo drug testing, six PDX models that exhibited stable engraftment and sufficient tumor growth through serial passages (F1–F4) were selected. These models were derived from patients with HNC, including several who had previously received platinum‐based chemotherapy. PDX tumor fragments (approximately 10 mm^3^) were transplanted into both flanks of BRJ mice (aged 6–10 weeks; at least 10 mice per model) following the same procedure as PDX passaging. When the tumors reached approximately 100–250 mm^3^ in volume, mice were randomly assigned to two groups. Cisplatin (CDDP) or control PBS was administered intraperitoneally on days 1 and 8, and tumor size was measured weekly using a digital caliper. Tumors were excised at the indicated time points for pathological analysis.

### 2.10 Statistical Analysis

All statistical analyses and data visualizations were performed using GraphPad Prism 10 (GraphPad Software). Fisher's exact test was used to evaluate the association between engraftment success and clinical variables. Tumor growth duration across PDX passages was compared using the Kruskal–Wallis test followed by Dunn's multiple‐comparisons test. Changes in tumor volume following CDDP treatment were analyzed using the Wilcoxon matched‐pairs signed‐rank test. A *p*‐value < 0.05 was considered statistically significant.

## 3 Results

### 3.1 Establishment of a Large‐Scale PDX Library for Head and Neck Cancer

We sought to establish a large‐scale PDX library for head and neck cancer (HNC). Each case was designated with the prefix “HN” (head and neck), followed by a unique numerical identifier. With informed consent, tumor specimens were obtained from over 100 patients diagnosed with HNC at the Department of Otolaryngology–Head and Neck Surgery, Fujita Health University Hospital. The residual tissues from surgical resections were used for PDX generation. Patient demographics and tumor histology are summarized in Tables 1 and 2. Oral cancer was the most common type (42%), followed by oropharyngeal (17%) and hypopharyngeal cancer (12%). Notably, our library includes 9 cases (approximately 10%) of salivary gland cancer.

### 3.2 Genomic Landscape of Patient Tumors

**FIGURE 1:**
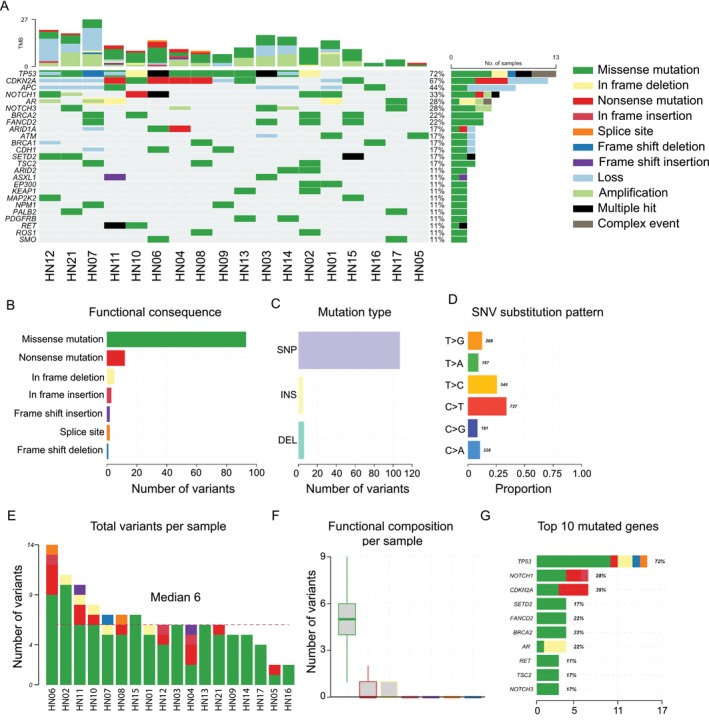
Gene profiling of patients enrolled in the PDX library. (A) Genetic alterations in cancer‐associated genes were analyzed using the *PleSSision‐Rapid* panel (145‐gene panel) in tumor specimens from 18 patients with HNC. The heatmap illustrates 11 types of genomic alterations, color‐coded accordingly. Stacked bar graphs above each column represent the tumor mutational burden (TMB). To the right of the heatmap, percentages indicate the proportion of samples (out of 18) harboring at least one alteration in each gene, and the adjacent stacked bar graph displays the frequency and composition of these alterations. (B–D) Summary of variant classifications and substitution patterns. (B) The number of variants classified by functional consequence. (C) The number classified by variant type. (D) Proportion of single‐nucleotide variant (SNV) substitution types. (E, F) Summary of variant counts per sample. (E) Total variant counts per tumor sample, with the red dashed line indicating the median across all samples (median = 6). (F) The distribution of variant counts per sample across functional consequence categories. (G) Distribution of all detected mutation events across the top 10 most frequently mutated genes. Percentages to the right indicate the fraction of all detected mutations occurring in each gene.

We first analyzed the genomic profiles of patients enrolled in our PDX library using the *PleSSision‐Rapid* panel, which targets 145 cancer‐related genes. In line with previous studies [5, 6], alterations in the tumor suppressor gene *TP53*—including mutations and deletions—were detected in over 70% of cases. Mutations in other tumor suppressor genes, such as *CDKN2A* and adenomatous polyposis coli (*APC*), were also observed at lower frequencies. In addition, mutations in the *NOTCH* gene family, which is involved in cellular differentiation, were identified in approximately 30% of patients. However, no activating mutations in major oncogenes were detected (Figure 1A). Functional classification of variants revealed that missense mutations were the most common type, and C > T transitions represented the predominant class of single‐nucleotide variants (Figure 1B–D). The number of variants per sample varied considerably among patients, with a median of 6 mutations per tumor (Figure 1E,F). When the distribution of all mutation events was summarized, *TP53*, *NOTCH1*, and *CDKN2A* emerged as the most frequently mutated genes (Figure 1G).

To assess the representativeness of our cohort, we compared the mutation frequencies of major genes with those reported in the Cancer Genome Atlas Head–Neck Squamous Cell Carcinoma (TCGA‐HNSC) dataset. The overall mutational spectrum was similar between the two cohorts, encompassing key driver genes such as TP53 (72.2% in our cohort vs. 71.5% in TCGA‐HNSC), CDKN2A (38.9% vs. 22.1%), NOTCH1 (27.8% vs. 17.8%), and PIK3CA (5.6% vs. 18.4%), with minor discrepancies likely reflecting differences in the targeted gene panel and cohort composition (Supplementary Table S1).

### 3.3 Engraftment Success and Associated Clinical Factors

**FIGURE 2:**
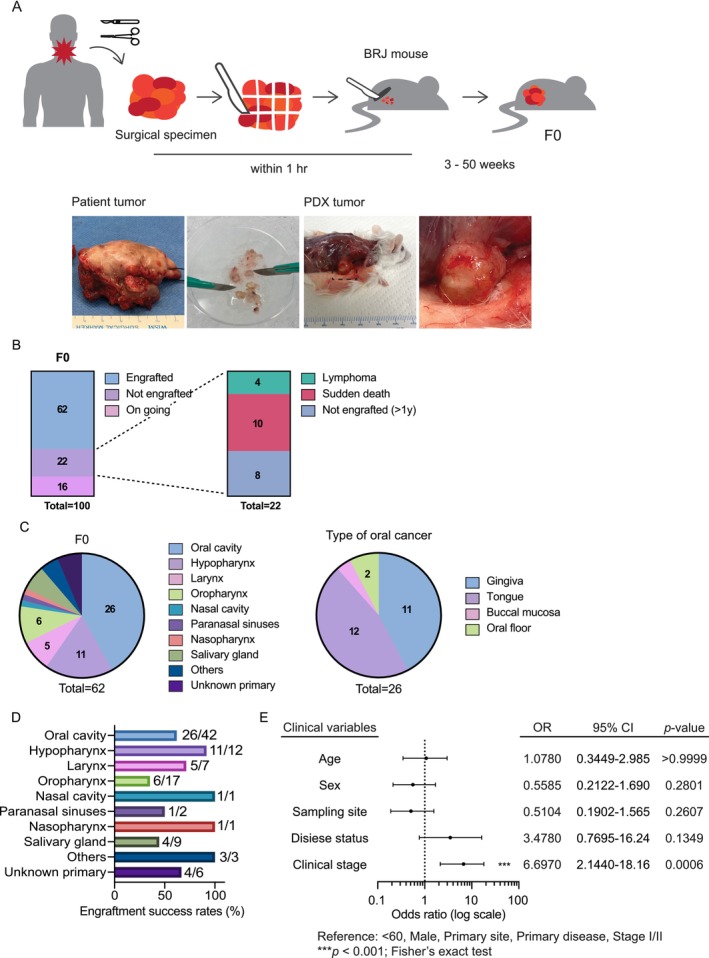
Generation of HNC PDX models. (A) Schematic overview of PDX generation. Tumor tissue resected from HNC patients was cut into 2–30 mm^3^ pieces and subcutaneously implanted into immunodeficient mice. (B) Summary of PDX engraftment outcomes (left) and reasons for engraftment failure (right) among 100 patient‐derived tumor transplantations. (C) Distribution of cancer types among tumors with 62 successful PDX engraftments. Oral cancer subsites are shown separately on the right. (D) Engraftment success rates by anatomical site (F0). Bars represent the percentage of successfully engrafted tumors for each anatomical site. Values to the right of each bar indicate the number of engrafted tumors relative to the number of implanted tumors (e.g., 26/42). Engraftment rates ranged from 35% in oropharyngeal cancers to over 90% in hypopharyngeal cancers. (E) Association between patient clinical characteristics and PDX engraftment. A forest plot illustrates the odds ratios (ORs) and 95% confidence intervals (CIs) for PDX engraftment according to age (< 60 vs. ≥ 60 years), sex (male vs. female), sampling site (primary vs. metastatic lesion), disease status (primary vs. recurrent), and clinical stage (I/II vs. III/IV). Fisher's exact test was used for statistical analysis. Only clinical stage showed a significant association with PDX engraftment (***p < 0.001).

To establish a comprehensive PDX library for HNC, we implemented a standardized protocol integrating several previously reported methodologies (see *Materials and Methods*, and visualized in Figure 2A). As of this analysis, tumor specimens from 100 patients had been transplanted. Of these, 62 successfully engrafted, 22 failed, and 16 remain under observation (Figure 2B). The failures were attributable to lymphoma outgrowth (*n* = 4), host mouse death (*n* = 10), or prolonged absence of tumor growth (> 1 year; *n* = 8). Among the successfully engrafted F0 tumors, oral cancers accounted for the largest proportion (approximately 42%), followed by hypopharyngeal (18%) and laryngeal cancers (8%) (Figure 2C), closely reflecting the overall tumor distribution in the cohort. We also compared engraftment success rates across anatomical sites (Figure 2D). The F0 engraftment rates varied among tumor types, ranging from 35% in oropharyngeal cancers to over 90% in hypopharyngeal cancers, with oral cavity cancers showing an intermediate success rate of approximately 62%. Although the number of cases was limited in several sites, hypopharyngeal tumors tended to engraft at a higher rate.

We next assessed associations between clinical variables and PDX engraftment using Fisher's exact test (Figure 2E). The variables analyzed included age (< 60 vs. ≥ 60 years), sex (male vs. female), sampling site (primary vs. metastatic lesion), disease status (primary vs. recurrent), and clinical stage (I/II vs. III/IV). Among these, only advanced clinical stage (III/IV) was significantly associated with successful engraftment (*p* < 0.001), whereas no other variables showed a significant correlation (all *p* > 0.1).

### 3.4 Serial Passaging and Tumor Growth Dynamics

**FIGURE 3:**
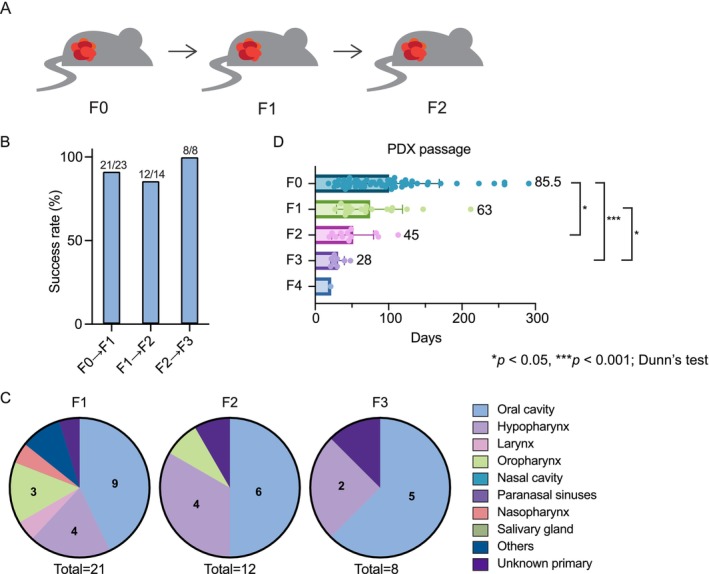
Sequential xenotransplantation of PDX tumors in immunodeficient mice. (A) Schematic overview of sequential PDX tumor xenotransplantation. Tumors established in mice from patient‐derived tissues (F0) were harvested at a tumor volume of 400–2000 mm^3^, cut into 2–30 mm^3^ pieces, and subcutaneously implanted into immunodeficient mice to generate the F1 generation. This process was repeated to generate subsequent passages (F2 and F3). (B) Engraftment success rates across serial passages (F0–F3). Bars represent the percentage of engrafted tumors per total attempted tumors. Values above the bars indicate the number of engrafted and total attempted tumors. The consistently high success rates (> 85%) confirm stable engraftment capacity across passages. (C) Distribution of cancer types among tumors with successful PDX engraftment (F1–F3). (D) Tumor growth duration across passages (F0–F4). The durations (in days) from implantation to the next passage are presented, with the median value shown to the right of each bar. Error bars indicate standard deviations (SD). Sample sizes: F0 (*n* = 62), F1 (*n* = 21), F2 (*n* = 12), F3 (*n* = 8), and F4 (*n* = 1). A Kruskal–Wallis test revealed a significant difference in growth duration across F0–F3 (H = 23.30, df = 3, *p* < 0.0001). Dunn's multiple comparisons test showed significant differences between F0 and F2 (*p < 0.05), F0 and F3 (***p < 0.001), and F1 and F3 (*p < 0.05). *p < 0.05, ***p < 0.001; Dunn's test following Kruskal‐Wallis analysis.

Tumors established in the F0 generation were serially passaged to evaluate tumor growth and engraftment capacity in later generations (Figure 3A). Of the 62 tumors in F0, 21 successfully engrafted to F1. Engraftment success rates remained consistently high across serial passages (91.3%, 85.7%, and 100% for F0 to F1, F1 to F2, and F2 to F3, respectively; Figure 3B), indicating stable tumor‐propagating ability of established PDXs. The apparent decrease in the total number of models reflected reduced re‐implantation attempts rather than engraftment failure. The anatomical distribution of successfully engrafted tumors in F1–F3 resembled that of F0, with oral cancers being the most prevalent, followed by hypopharyngeal cancers (Figure 3C).

The median duration to reach the next passage progressively decreased from F0 to F3: 85.5 days for F0, 63 days for F1, 45 days for F2, and 28 days for F3. Only one tumor was available at F4, which exhibited the shortest outgrowth time. A Kruskal–Wallis test revealed a significant difference in growth duration across F0–F3 (*p* < 0.0001). Post hoc Dunn's tests showed that F2 and F3 tumors grew significantly faster than those in F0, and that F3 tumors also grew significantly faster than those in F1. These findings suggest that later‐generation PDX tumors exhibit accelerated growth, possibly due to adaptation to the murine host and selection of faster‐growing clones.

### 3.5 Genomic Comparison Between Patient Tumors and PDX Tumors

**FIGURE 4:**
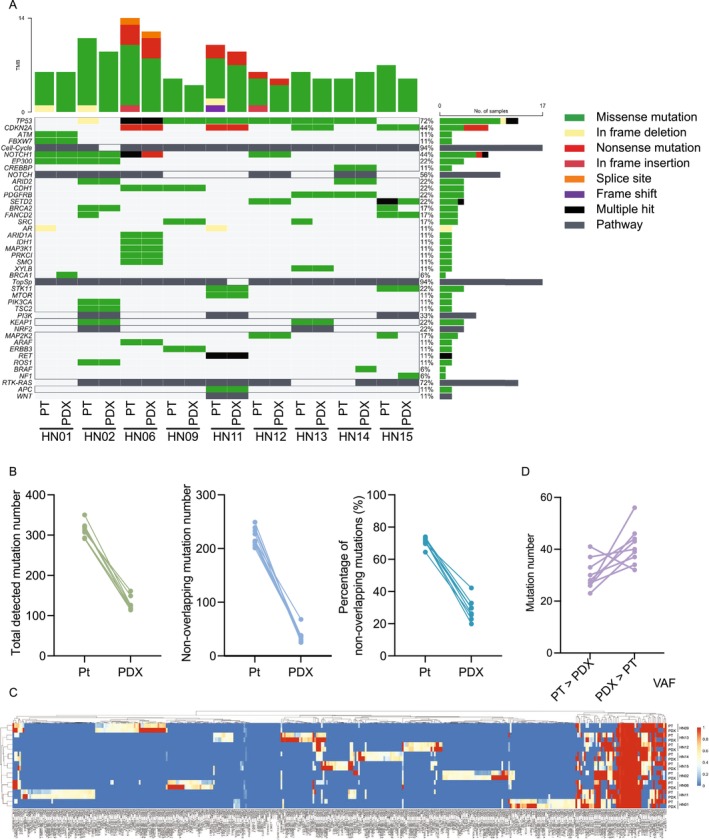
Comparison of gene mutations between patient tumors and PDX tumors. All panels compare patient tumors and their corresponding PDX tumors across multiple genomic parameters. (A) Genetic alterations were analyzed using the *PleSSision‐Rapid* panel in 9 patient‐PDX tumor pairs. The heatmap displays genomic alterations in selected cancer‐associated genes, color‐coded by alteration type. Stacked bar graphs above each column indicate the number and types of alterations contributing to the tumor mutation burden (TMB). The horizontal bar graph on the right shows the frequency and composition of alterations observed in each gene across all samples. (B) Comparison of total detected mutations (left), non‐overlapping mutation numbers (center), and the percentage of non‐overlapping mutations (right) between patient tumors and PDX tumors. Each line connects a matched patient–PDX pair. PDXs exhibited fewer total and non‐overlapping mutations than their corresponding patient tumors, suggesting loss of some patient‐specific mutations and the emergence of a smaller number of PDX‐specific mutations during engraftment. (C) Variant allele frequencies (VAFs) for all detected mutations across 9 patient–PDX pairs. Columns indicate mutations annotated with gene, nucleotide position, and base change, while rows represent paired tumors. Color intensity reflects VAF values. (D) Number of mutations with altered VAFs in 9 patient–PDX pairs. In 8 of 9 cases, more mutations had increased VAFs in PDXs, suggesting selective expansion of specific subclones in the PDX environment.

To assess potential genomic drift during engraftment, we performed targeted sequencing using the gene panel on F2‐generation PDX tumors and their matched patient tumors (*n* = 9). Among the 12 F2 tumors established, 9 were successfully sequenced, whereas 3 were excluded due to insufficient tissue or incomplete engraftment. In 8 out of 9 cases, the tumor mutation burden (TMB), defined as the number of mutations per megabase (Mb) of the sequenced genome, was reduced in the PDX tumors compared with the original patient tumors (Figure 4A). The major mutational patterns in the frequently altered genes were maintained in the PDX tumors, indicating that key genomic alterations were largely preserved. The total number of detected mutations decreased to approximately one‐third (Figure 4B, left). Non‐overlapping mutation profiles revealed that a large number of mutations present in patient tumors were lost during engraftment, whereas only a small number of newly emerged PDX‐unique mutations were observed (Figure 4B, center and right). Consistent with this pattern, the analysis of variant allele frequencies (VAFs) demonstrated that, in 8 of 9 pairs, a greater number of mutations exhibited increased VAFs in the PDX tumors (Figure 4C,D). These findings collectively indicate clonal selection and evolutionary dynamics during xenograft establishment.

We classified detected variants into three categories: patient‐unique, patient–PDX common, and PDX‐unique mutations. Based on the OncoKB Cancer Gene List, genes were annotated as oncogenes or tumor suppressor genes. The distribution of driver genes was comparable among categories, indicating no selective enrichment of drivers in PDX‐unique mutations (Figure S1 and Table S2). Consistent with previous findings [27], these results indicate that PDX tumors undergo selective pressure in the murine host, resulting in the expansion of specific tumor subclones and the emergence of PDX‐specific mutations, while core genomic alterations remain largely conserved.

To examine potential selection pressures during xenograft establishment, we estimated the dN/dS ratios (nonsynonymous/synonymous substitution rates) for all coding mutations in each tissue–PDX pair using the dndscv package. The log_10_‐transformed MLE values (log_10_[MLE + 1e–3], where 1e–3 was added to avoid log‐zero values) were compared between patient tumors and PDX tumors for each mutation category (all coding, missense, nonsense, splice‐site, and truncating mutations). No significant differences were observed across any mutation type, suggesting that no consistent evidence of directional selection was detected during PDX engraftment (Figure S1).

### 3.6 Histopathological and Stromal Features of PDX Tumors

**FIGURE 5:**
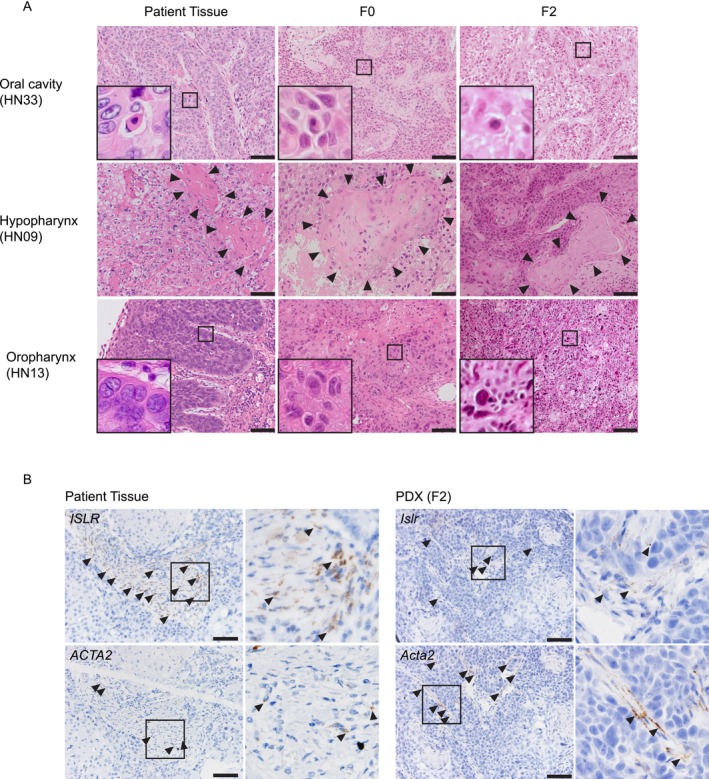
Pathological comparison between patient tumors and PDX tumors. (A) Representative HE‐stained sections of patient primary tumors (left), PDX tumors at F0 (middle), and F2 (right) are shown. Individual cell keratinization in HN33 (top row), areas of keratinization in HN09 (middle row), and nuclear pleomorphism in HN13 (bottom row) are highlighted. Key histopathological features were maintained throughout PDX engraftment and serial passaging. Scale bars, 100 μm. (B) In situ hybridization (ISH) analysis of CAFs in patient tumors (left) and matched PDX tumors at passage F2 (right) from HN09 (hypopharyngeal cancer). CAF subtype markers: ISLR (rCAF, top) and ACTA2 (myCAF, middle). Arrowheads indicate ISH‐positive stromal cells. Rectangular regions were enlarged to the right. Stromal CAF subtype–specific marker expression patterns were retained following PDX engraftment and passaging, but their proportions were altered. Scale bars, 100 μm.

PDX models are thought to retain the pathological characteristics of the original patient tumors [28]. To assess this in our HNC PDXs, we compared histological features between patient tumors and their corresponding PDX tumors (F0 and F2) (Figure 5A). In HN33 (oral cancer), focal keratinization at the single‐cell level was maintained across generations. In HN09 (hypopharyngeal cancer), well‐differentiated morphology and keratin pearls were preserved. In HN13 (oropharyngeal cancer), nuclear pleomorphism and dyskeratosis were consistently observed. These findings indicate that key histopathological features are maintained during PDX propagation.

Given that stromal components in PDXs are replaced by murine cells [23], we further examined the expression of cancer‐associated fibroblast (CAF) markers using RNA in situ hybridization (ISH) with *ISLR* (a marker of tumor‐restraining CAFs, rCAFs) and *ACTA2* (a marker of tumor‐promoting CAFs, pCAFs) in patient and matched PDX tumors (Figure 5B). In patient tumors, both *ISLR* and *ACTA2* showed stromal staining. These patterns were largely preserved in PDX tumors, but a decrease in *ISLR*‐positive signals accompanied by an increase in *ACTA2*‐positive signals was observed, suggesting a shift in the CAF composition. These results suggest that although CAF‐like populations persist in PDX models, stromal remodeling occurs, warranting caution when using PDXs to study tumor–stroma interactions.

### 3.7 Drug Response in PDX Models

**FIGURE 6:**
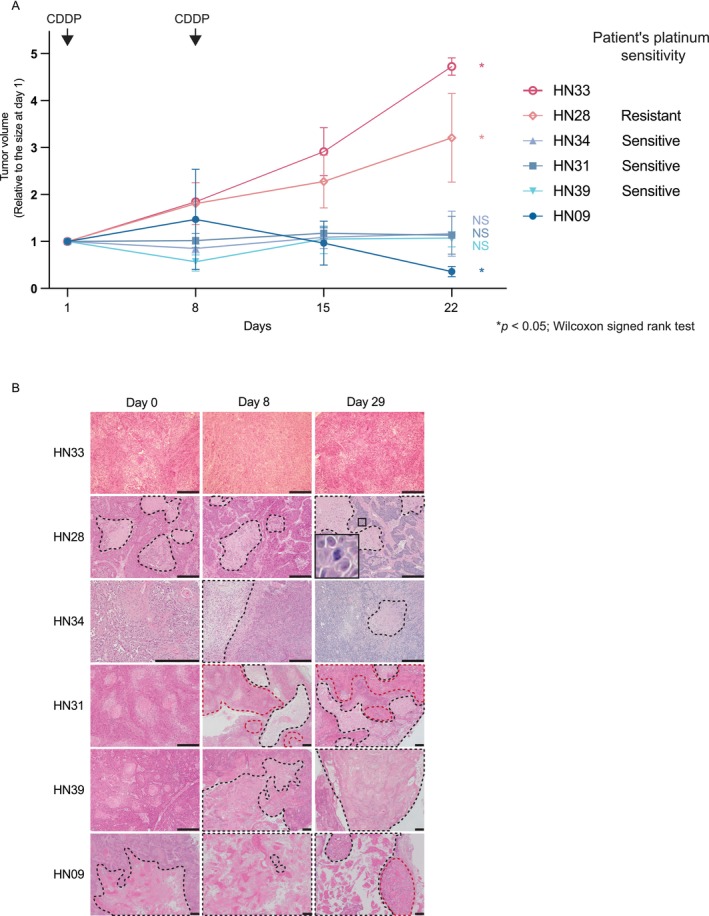
Conserved drug sensitivity of patients in PDX tumors. (A) Effects of cisplatin (CDDP) treatment on tumor growth in PDX models. PDX tumors were treated with CDDP (5 mg/kg, intraperitoneally) on days 1 and 8. Tumor volumes were measured and plotted as relative values normalized to day 1. Clinical CDDP sensitivity was defined as no progression within six months after platinum‐based chemotherapy. PDXs derived from clinically sensitive patients (HN34, HN31, HN39) showed no significant change in tumor volume, whereas the model from a resistant patient (HN28) showed significant growth (*p* < 0.05). HN33 showed significant growth (p < 0.05), and HN09 showed regression (*p < 0.05); sensitivity status of these two patients was unknown. Data represent mean ± SD from more than three independent experiments. *p < 0.05 vs. day 1; Wilcoxon signed‐rank test. (B) HE‐stained sections of PDX tumors collected on days 0, 8, and 29 following CDDP administration. Necrotic areas are outlined in black dotted lines, and keratinizing regions in red. HN28 (resistant) exhibited atypical mitoses on day 29 (inset), suggesting high proliferative activity. HN09 (regressive) and tumors from sensitive patients (HN34, HN31, HN39) showed increased necrosis and keratinization, whereas HN33 showed no notable histological changes. Scale bars, 300 μm.

Finally, we evaluated the utility of our PDX platform for assessing drug sensitivity using cisplatin (CDDP), a standard chemotherapeutic agent for HNC. CDDP was administered intraperitoneally on days 1 and 8, and tumor volumes were monitored over time (Figure 6A). Based on the growth response, PDX models were categorized as CDDP‐sensitive (HN09, HN31, HN34, HN39) or ‐resistant (HN28, HN33). These responses were consistent with clinical CDDP sensitivity of the original patients: HN31, HN34, and HN39 from sensitive patients, HN28 from a resistant patient. HN09 and HN33 lacked clinical treatment history.

To further characterize drug effects, we performed pathological analysis of the PDX tumors treated with CDDP (Figure 6B). CDDP‐sensitive PDX tumors exhibited increased necrosis and keratinization, whereas resistant PDX tumors showed minimal change. Notably, HN28 PDX tumors treated with CDDP displayed atypical mitoses, indicative of ongoing proliferative activity despite treatment. These histological changes mirrored the tumor volume responses, supporting the translational relevance of our PDX models.

We also explored whether cisplatin sensitivity could be linked to specific gene alterations detected in our panel sequencing data. No consistent or statistically significant associations were identified, suggesting that CDDP response in HNC may depend on broader molecular and phenotypic factors rather than gene mutations.

## 4 Discussion

In the present study, we successfully established a large‐scale PDX library, designated the Fujita Xenograft Library (FXeL), which is specifically focused on HNC—a malignancy characterized by limited therapeutic options and generally poor clinical outcomes. By leveraging an optimized engraftment protocol with high efficiency, we were able to generate a comprehensive collection of PDX models annotated with detailed clinical information that faithfully recapitulates the biological characteristics of the original patient tumors. Accordingly, FXeL represents a robust and versatile preclinical platform for basic research and FPO in HNC.

Precision oncology has increasingly sought to complement static and descriptive measurements with functional assays, termed FPO, for several important reasons [4, 8, 9]. First, several cancers, such as HNC, often lack actionable oncogenic driver mutations. Second, only a small population of patients can benefit from available treatment options. Third, patients with identical genetic mutations may display diverse responses to the same targeted therapy. In this context, FXeL represents a powerful, robust, and scalable preclinical platform for FPO. Not only is it linked to detailed clinical information, but it also enables stratification of HNC patients based on comprehensive molecular and phenotypic profiling as well as the results of functional assays. Such integration allows for the identification of novel therapeutic targets and tumor vulnerabilities that may not be apparent through genomic analysis alone.

Furthermore, FXeL can be utilized to develop patient‐derived tumor organoids, which offer advantages such as rapid turnaround time, high scalability, and suitability for high‐throughput applications [10, 11]. Indeed, we have successfully generated more than 30 tumor organoid lines from FXeL. Moreover, FXeL holds broad applicability across multiple research domains, including preclinical evaluation of investigational agents, prediction of clinical trial responses, and discovery of therapeutic targets for rare cancers. Taken together, this FXeL resource is expected to significantly advance HNC research and facilitate the development of more effective, biology‐driven treatment strategies in the era of FPO.

The tumor microenvironment has a major impact on tumor progression and therapeutic response [29]. Since PDX models generally require immunodeficient hosts (mice, in this study) for their generation, they inherently lack human tumor microenvironmental components, including immune cells, thereby precluding the investigation of tumor–immune cell interactions. Moreover, human stromal elements are gradually replaced by murine counterparts. Consistent with this, we observed alterations in the composition of CAFs. To address these shortcomings, humanized PDX models have been developed, in which human immune cell populations are reconstituted in immunocompromised mice, using blood from the same patients as the tumors [30, 31], tumor‐infiltrating lymphocytes derived from the same tumor tissues [32], or engineered human thymus organoids generated from induced pluripotent stem cells [33]. Future studies incorporating human immune compartment into PDX models are highly anticipated and will further expand the applications of PDX models in FPO.

Although several limitations of PDX models have been recognized, various strategies to overcome these challenges have also been proposed. We believe that PDX models will play an increasingly important role in FPO. In particular, the integration of rapidly advancing artificial intelligence technologies with PDX‐based FPO holds great promise for identifying previously unrecognized tumor vulnerabilities, discovering novel therapeutic strategies, and uncovering new biomarkers that may predict treatment response and patient prognosis.

## Author Contributions

**Hisano Yanagi:** conceptualization (equal), data curation (equal), formal analysis (equal), funding acquisition (equal), investigation (equal), methodology (equal), project administration (equal), writing – original draft (equal). **Tomoki Kuki:** investigation (equal), resources (equal). **Tetsuya Takimoto:** data curation (equal), formal analysis (equal), investigation (equal). **Miki Takabayashi:** data curation (equal), formal analysis (equal), investigation (equal). **Seiji Yamada:** investigation (equal). **Chikako Yagi:** data curation (equal), formal analysis (equal), investigation (equal), resources (equal). **Yoshitake Kiryu:** data curation (supporting), investigation (supporting), resources (equal). **Maki Kurimoto:** investigation (supporting). **Miho Ishikawa:** investigation (supporting). **Arisa Yoshida:** investigation (supporting). **Yasuyoshi Mizutani:** resources (equal). **Atsushi Enomoto:** investigation (supporting), supervision (supporting). **Motoshi Suzuki:** supervision (supporting). **Kenji Kawada:** supervision (supporting). **Hisayuki Kato:** conceptualization (supporting), funding acquisition (equal), investigation (equal), methodology (equal), project administration (equal), resources (equal), supervision (supporting). **Ichiro Tateya:** supervision (lead). **Hideyuki Saya:** conceptualization (equal), funding acquisition (equal), project administration (equal), supervision (lead), writing – original draft (equal). **Takashi Watanabe:** conceptualization (equal), funding acquisition (equal), investigation (equal), methodology (equal), project administration (equal), writing – original draft (equal).

## Funding

This study was supported by Grants‐in‐Aid for Scientific Research (KAKENHI) from the Ministry of Education, Culture, Sports, Science and Technology of Japan (23K15897 to H.Y., 23K08948 to H.K., 23K06660 to T.W., and 23H00410 to H.S.), and by the Takeda Science Foundation (to T.W.).

## Ethics Statement

Approval of the research protocol by an Institutional Review Board: Ethics Committee of Fujita Health University (Approval No. HM24‐224). Animal Studies: Approved by the Experimental Animal Committee at Fujita Health University (Approval No. APU22132‐MD7‐CH1).

## Consent

The authors have nothing to report.

## Conflicts of Interest

The authors declare no conflicts of interest.

## Supporting information


**Figure S1:** Genomic dynamics and selection pressure during PDX establishmentA. Distribution of oncogenes and tumor suppressor genes among variant categories. Bar plots show the distribution of gene functional classifications (oncogene, tumor suppressor gene, both, or insufficient evidence) across four variant categories: PDX‐unique, patient tissue–unique, partly lost in PDX, and common mutations. The total number of variants in each category is indicated below each bar (Total = 61, 89, 29, and 96, respectively). Gene classifications were based on annotations in the OncoKB Cancer Gene List.B. Comparison of dN/dS ratios between patient tumors and matched PDX tumors. Box plots show the estimated dN/dS ratios (nonsynonymous to synonymous substitution rates) for all coding, missense, nonsense, splice‐site, and truncating mutations in each paired patient tumor and matched PDX tumor. Values represent the log_10_‐transformed maximum likelihood estimates [log_10_(MLE + 1e–3)], as calculated using the dndscv package. Each line connects a matched Tissue–PDX pair. No significant differences were observed between patient tumors and PDX tumors across any mutation category, suggesting no consistent directional selection during PDX engraftment.

**Table S1:** Comparison of mutation frequencies with TCGA‐HNSC Comparison of mutation frequencies of representative cancer‐related genes between this cohort and the TCGA Head and Neck Squamous Cell Carcinoma (TCGA‐HNSC) dataset.

**Table S2:** Classification of genomic variants in patient tumors and PDX tumors. List of genomic variants detected in patient tumors and their matched PDX tumors, including gene name, protein change, driver status, gene type based on the OncoKB Cancer Gene List, occurrence across samples, and variant category (PDX‐unique, patient tissue–unique, common, or partly lost in PDX).

**Table S3:** List of mutation calls for patient tumors and PDX tumors. List of mutation calls derived from targeted sequencing of patient tumors and their matched PDX tumors, including genomic coordinates, variant classification, allele information, read depth, and functional annotations.

## Data Availability

Due to patient privacy and ethical restrictions, the raw sequencing data generated in this study are not publicly available. All processed mutation call data are provided in Table S3. Additional information is available from the corresponding author upon reasonable request.
