# Supplementary figures and images for: Establishment of a Large‐Scale PDX Library of Head and Neck Cancers for Functional Precision Oncology
Source: Cancer Med. 2026 Feb 18;15(2):e71521. doi: 10.1002/cam4.71521 (PMC12916448; doi:10.1002/cam4.71521)

Supplemental Figure S1 Genomic dynamics and selection pressure during PDX establishment

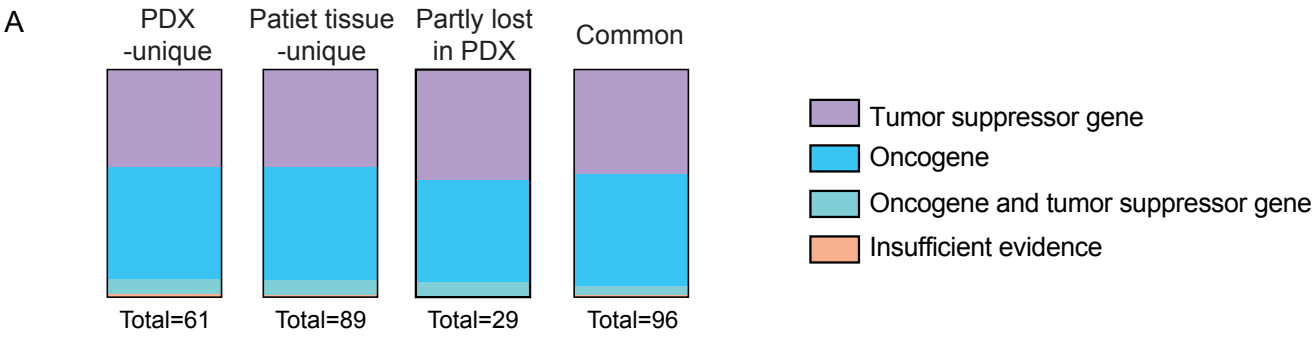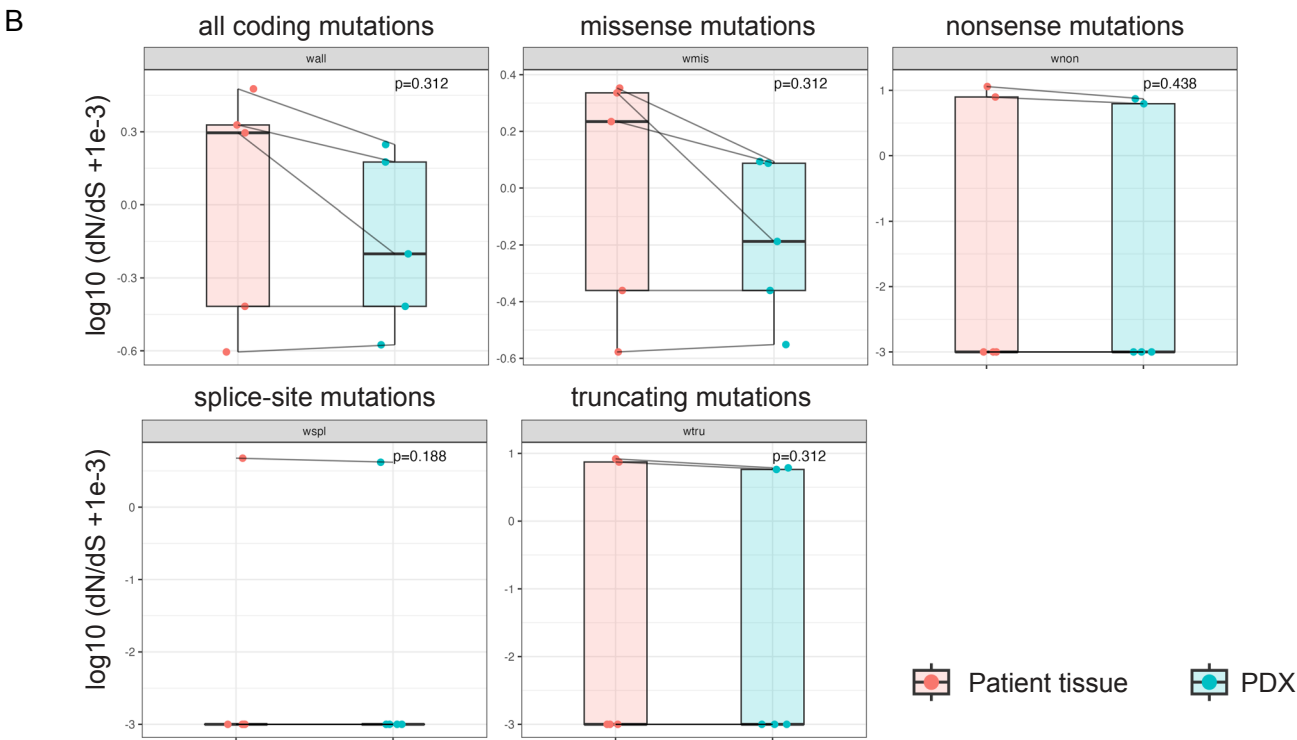

Supplement: Supplementary file 1 — Figure S1: Genomic dynamics and selection pressure during PDX establishmentA. Distribution of oncogenes and tumor suppressor genes among variant categories. Bar plots show the distribution of gene functional classifications (oncogene, tumor suppressor gene, both, or insufficient evidence) across four variant categories: PDX‐unique, patient tissue–unique, partly lost in PDX, and common mutations. The total number of variants in each category is indicated below each bar (Total = 61, 89, 29, and 96, respectively). Gene classifications were based on annotations in the OncoKB Cancer Gene List.B. Comparison of dN/dS ratios between patient tumors and matched PDX tumors. Box plots show the estimated dN/dS ratios (nonsynonymous to synonymous substitution rates) for all coding, missense, nonsense, splice‐site, and truncating mutations in each paired patient tumor and matched PDX tumor. Values represent the log10‐transformed maximum likelihood estimates [log10(MLE + 1e–3)], as calculated using the dndscv package. Each line connects a matched Tissue–PDX pair. No significant differences were observed between patient tumors and PDX tumors across any mutation category, suggesting no consistent directional selection during PDX engraftment. [file CAM4-15-e71521-s003.pdf]
